# Enhanced Activation of Rac1/Cdc42 and MITF Leads to Augmented Osteoclastogenesis in Autosomal Dominant Osteopetrosis Type II

**DOI:** 10.1002/jbm4.10070

**Published:** 2018-07-16

**Authors:** Youn‐Kwan Jung, Ki‐Tae Kwon, Ji‐Ae Jang, Min‐Su Han, Gun‐Woo Kim, Seungwoo Han

**Affiliations:** ^1^ Laboratory for Arthritis and Bone Biology Fatima Research Institute Daegu Fatima Hospital Daegu Republic of Korea; ^2^ Department of Internal Medicine Kyungpook National University Hospital Daegu Republic of Korea; ^3^ Department of Internal Medicine Daegu Fatima Hospital Daegu Republic of Korea

**Keywords:** AUTOSOMAL DOMINANT OSTEOPETROSIS, CHLORIDE CHANNEL 7, OSTEOCLAST DIFFERENTIATION, BONE RESORPTION

## Abstract

The autosomal dominant osteopetrosis type II (ADOII) caused by the mutation of chloride channel 7 (ClC‐7) gene is the most common form of adult‐onset osteopetrosis. Despite dysfunctional bone resorption, an augmented osteoclast differentiation was reported recently in ADOII patients. DNA sequencing analysis of the ADOII patient's *ClC‐7* gene identified a known heterozygous mutation, c.643G>A in exon 7, encoding p.Gly215Arg. In vitro osteoclast differentiation from the ADOII patient's peripheral blood mononuclear cells (PBMCs) increased compared with control despite their dysfunctional bone resorbing capacity. Osteoclasts from the ADOII patient's PBMCs and *ClC‐7* knockdown bone marrow monocytes (BMMs) showed an enhanced Ser‐71 phosphorylation of Rac1/Cdc42 and increase of the microphthalmia‐associated transcription factor (MITF) and receptor activator of NF‐κB (RANK) that can be responsible for the enhanced osteoclast differentiation. © 2018 The Authors. *JBMR Plus* published by Wiley Periodicals, Inc. on behalf of the American Society for Bone and Mineral Research.

## Introduction

Osteopetrosis is a group of inherited disorders in which the bones become harder and denser resulting from the dysfunction of osteoclasts.^1^ The most common and adult‐onset form with benign nature refers to the autosomal dominant osteopetrosis type II (ADOII), also known as Albers‐Schenberg disease.^2^ The molecular mechanism of ADOII is known as mutations in the voltage‐gated chloride channel 7 (*ClC‐7*) gene in chromosome 16p13.^1^

The acidification under the osteoclast attached to the bone surface is necessary to dissolve the crystallized calcium phosphate.^3^ It is mediated by an active transport of protons over the ruffled border membrane, which is driven by osteoclast V‐type H^+^ ATPase.^4^ At the same time, chloride ions are passively transported through chloride channels, mainly ClC‐7, to maintain the electrical neutrality, which is critical to keep the proton pump function and maintain the lacunae's highly acidic environment.^4^, ^5^ Reduced expression or function of ClC‐7 in osteoclast leads to the defect in the acidification of the resorption lacunae and consequently to failure of bone resorption.^6^, ^7^ The interesting pathologic feature in ADOII is a paradoxical increase of osteoclast number in vivo and in vitro despite their dysfunctional bone resorption.^8^, ^9^, ^10^, ^11^

Here we present a case of ADOII with the missense mutation of c.643G>A encoding p.Gly215Arg in exon 7 of *ClC‐7* gene. In spite of dysfunctional resorption capacity, in vitro osteoclast differentiation from peripheral blood mononuclear cells (PBMCs) was enhanced in the ADOII patient, and it was associated with enhanced phosphorylation of the small GTPases Rac1/Cdc42 and increase of the microphthalmia‐associated transcription factor (MITF) and receptor activator of NF‐κB (RANK).

## Patient and Methods

A 60‐year‐old Korean man was admitted complaining of right anterior chest pain after chest contusion, and radiographic evaluation revealed multiple fractures at the right 7th through 10th ribs. He had a number of past histories of fracture, including right distal radius, right humerus, right ankle, left 4th metatarsal, and ribs. He had hypertension and type 2 diabetes mellitus. His parents or children did not have a distinguished history of fracture. General examination revealed a normal stature of 170 cm, and he wore dentures because of dental caries and periodontitis.

**Figure 1:**
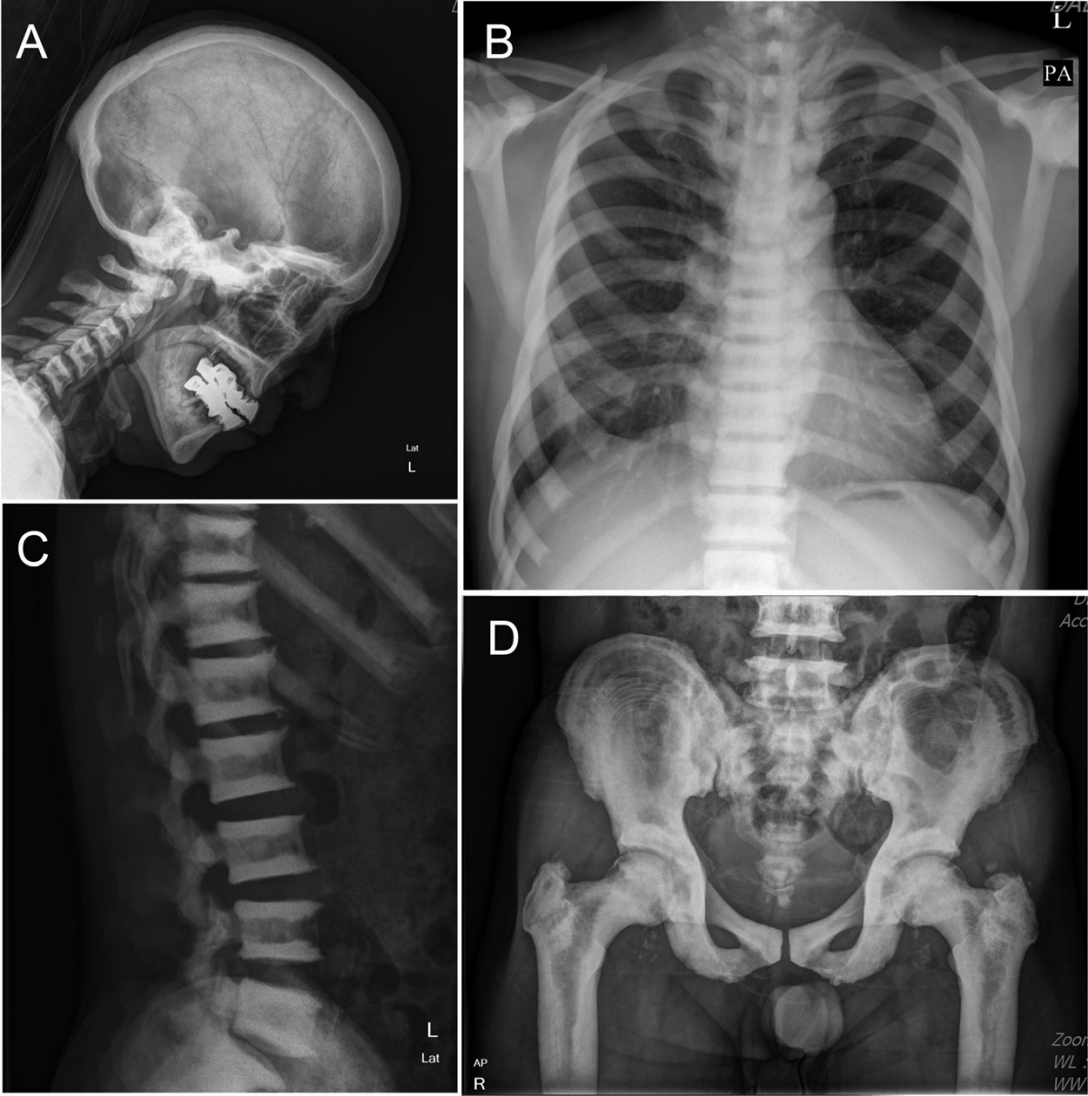
Skeletal radiographs reveal generalized symmetric increases in bone mass. (*A*) The cranium shows a thickening of the skull base. (*B*) Chest postero‐anterior radiograph shows the stark visualization of the anterior ribs. (*C*) Vertebral body shows a typical endplate sclerosis, called a “sandwich spine.” (*D*) Iliac crest also shows sclerotic and lucent bands, and the femoral bone shows cortical thickening and medullary encroachment.

Initial laboratory investigation revealed serum calcium of 8.5 mg/dL (reference level 9.1–11.0), serum phosphorus 3.3 mg/dL (2.3–4.7), serum intact parathyroid hormone 32.0 pg/mL (12.0–88.0), bone‐specific alkaline phosphatase 11.8 µg/L (11.6–20.10), and 25‐hydroxy vitamin D 27.7 ng/mL (30.1–100.0). The rest of his laboratory results including complete blood count, liver function tests, urinalysis, and other serologic tests were within normal limits. The chest radiograph revealed a generalized symmetric increase in bone mass, which showed a stark visualization of the anterior ribs. The skull radiograph showed a thickening of the base of skull. Alternating sclerotic and lucent bands were found in the iliac crests and in vertebral bodies, called “sandwich spine.” The femoral bone shows cortical thickening and medullary encroachment (Fig. 1). Lumbar spine bone mineral density (BMD) examined by dual‐energy X‐ray absorptiometry scan revealed a strikingly high *T*‐score of +11.8 at L_3_ and +11.6 at L_4_ level, and BMD of femoral head also increased to *T*‐score +8.8. The bone resorption marker of serum type I collagen C‐telopeptide (CTX) level was 0.167 ng/mL, which was in a lower normal range.

With the impression of ADOII, we analyzed the DNA sequence of coding region in the *ClC‐7* gene. And we conducted functional analysis during in vitro osteoclast differentiation from PBMCs, which was compared with that of two age‐ and sex‐matched controls with no underlying disease except hypertension. The detailed experimental methods are described in the Supplemental Methods. This study was approved by the ethics board of the Daegu Fatima Hospital, and patient consent was obtained.

## Results

### DNA sequencing of ClC‐7 gene in the ADOII patient reveals a missense mutation, G215R

**Figure 2:**
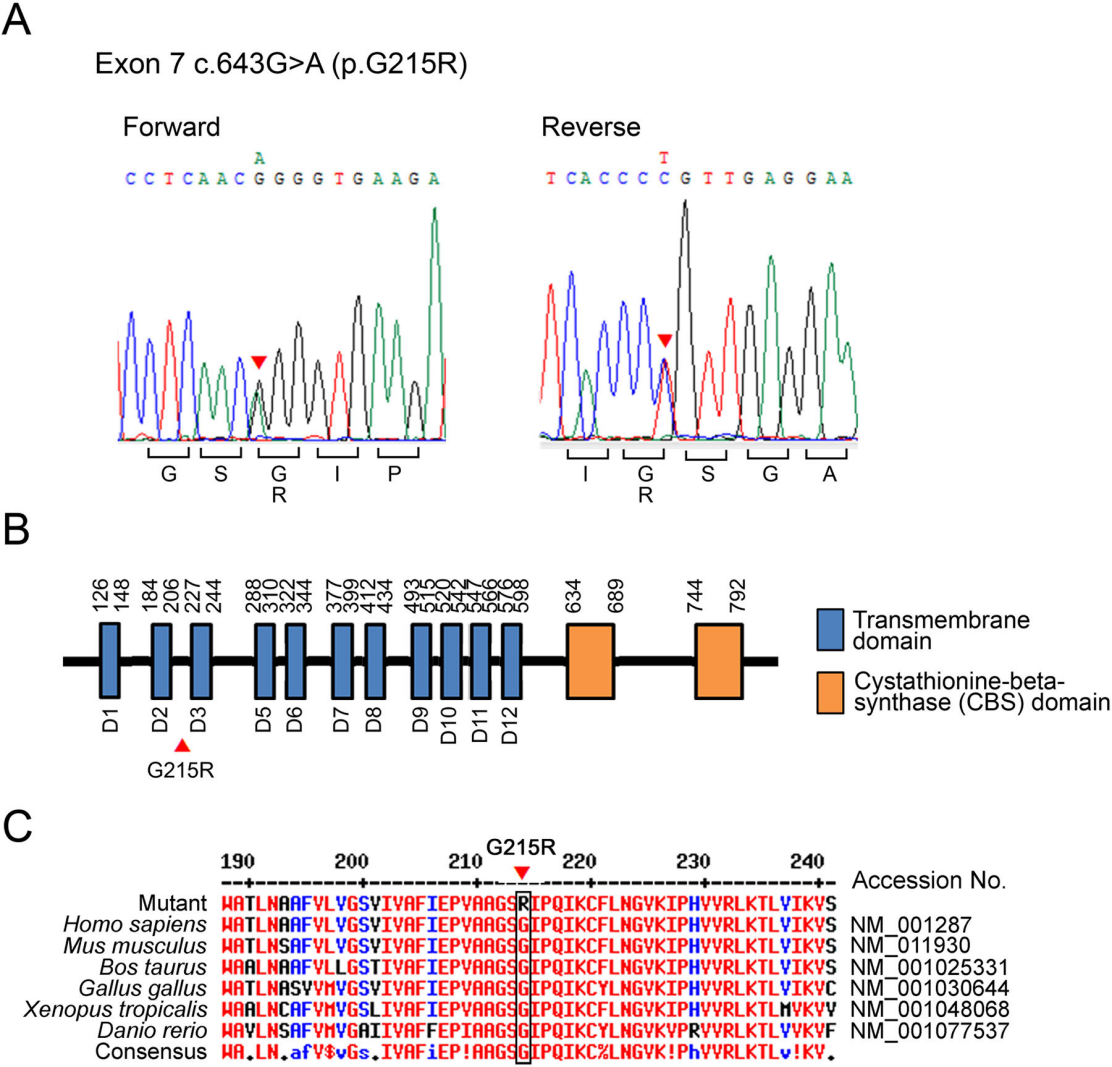
DNA sequencing identifies a missense mutation in the *ClC‐7* gene. (*A*) Heterozygous mutation of c.643G>A encoding p.Gly215Arg in exon 7 of *ClC‐7* gene was detected in the ADOII patient. (*B*) A schematic presentation of the ClC‐7 protein with the relative location of functional domains. The mutation G215R is located intracellularly between transmembrane domain 2 and 3. (*C*) Multiple sequence alignment of ClC‐7 protein in different species shows the phylogenetic conservation of Glycin at amino acid position 215.

The DNA sequencing analysis of the patient's *ClC‐7* gene identified a known heterozygous missense mutation, c.643G>A (NM_001287.5) in exon 7 (Fig. 2 *A*). This mutation results in the change of 215th amino acid of ClC‐7, Glycin into Arginin (p.Gly215Arg: NP_001278.1), which is located between transmembrane domain 2 and 3, where it is known to influence the channel's pore properties^12^ (Fig. 2 *B*). Multiple sequence alignment of ClC‐7 protein showed the phylogenetic conservation of Glycin at amino acid position 215 between different species (Fig. 2 *C*).

### Osteoclast differentiation from the ADOII patient's PBMCs is enhanced despite their dysfunctional bone resorbing capacity

**Figure 3:**
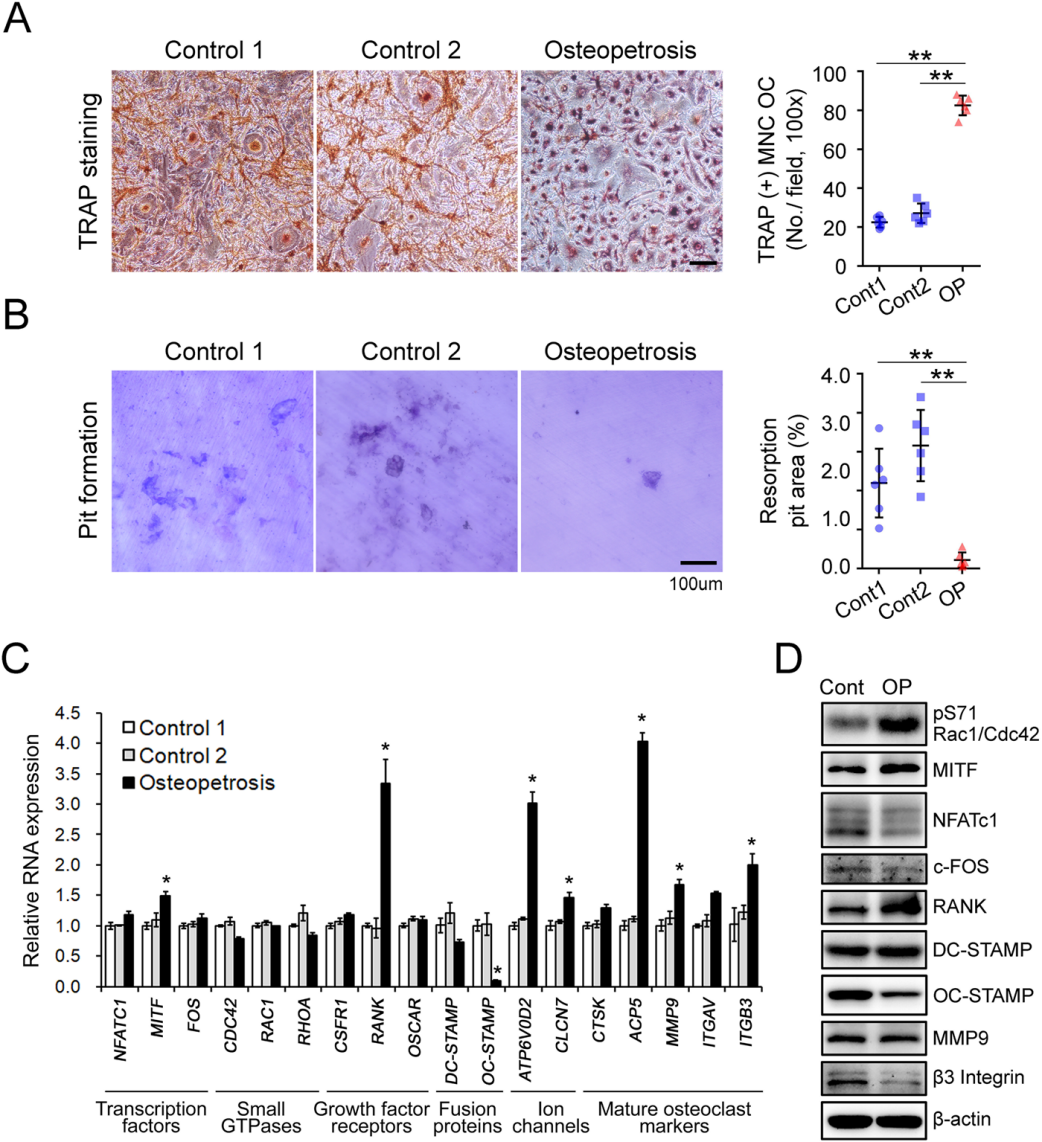
Osteoclast differentiation from PBMCs was enhanced in the ADOII patient, which can be dependent on the enhanced phosphorylation of small GTPase Rac1/Cdc42 and the expression of MITF and RANK. (*A*) Osteoclastogenesis from PBMCs of control and ADOII patient was assessed by TRAP staining. PBMCs were cultured with 30 ng/mL M‐CSF and 30 ng/mL RANKL for 21 days and then subjected to TRAP staining. TRAP^+^ multinuclear cells (MNCs) with more than three nuclei were counted as osteoclasts. **p < 0.01 compared with control. (*B*) Osteoclast function was assessed with pits formation assay. PBMCs were cultured on dentin slice with M‐CSF and RANKL for 21 days. The cells were washed and the resorption pits were stained with 1% toluidine blue and the area of pits were analyzed with Nikon's NIS‐Elements imaging software. Scale bar = 100 µm. (*C*) The alteration of major osteoclast gene expression in osteoclasts from the ADOII patient was assessed by real‐time qPCR. (*D*) Protein levels showed significant difference in RNA expression and those supposed to have a mechanical significance were validated by Western blot analysis.

To investigate the osteoclast differentiation capacity in the ADOII patient, PBMCs were cultured with 30 ng/mL RANKL and 30 ng/mL M‐CSF for 21 days. The PBMCs from the ADOII patient gave rise to significantly increased numbers of TRAP‐positive multinuclear osteoclasts compared with controls (Fig. 3 *A*). Despite the increase in the number of osteoclasts, the osteoclasts from the ADOII patient created less resorption area than in controls (Fig. 3 *B*).

### Enhanced phosphorylation of Rac1/Cdc42 and increase of MITF and RANK expression in osteoclasts from the ADOII patient

To elucidate the mechanism of enhanced osteoclastogenesis in ADOII, we screened the expression of major genes regulating osteoclast differentiation. Among three major transcription factors of osteoclast, the RNA expression of *MITF* was significantly increased compared with controls. In addition, the RNA expression of *RANK*, *ATP6V0D2*, and *ClC‐7* was increased in ADOII, but *CSFR1, OSCAR*, and *DC‐STAMP* was not. The RNA expression of small GTPases such as *CDC42*, *RAC1*, and *RHOA* was not different with controls, and mature osteoclast markers such as *ACP5* (*TRAP*), *MMP9*, and *ITGB3* were increased in ADOII. On the other hand, the expression of *OC‐STAMP* was significantly decreased in ADOII osteoclasts compared with controls (Fig. 3 *C*).

Despite there being no difference in RNA level, the CDC42, a small GTPase of the Rho family, is known to be activated by increase of intracellular chloride in primary ciliogenesis.^13^ The phosphorylation of RAC1/CDC42 was significantly increased in ADOII osteoclasts compared with control. Among transcription factors, the MITF protein level as well as mRNA increased in ADOII, whereas the protein level of NFATc1 and c‐FOS decreased in ADOII. The protein level of RANK also increased in osteoclasts from the ADOII patient. The fusion protein OC‐STAMP was attenuated in ADOII at the protein level, whereas the DC‐STAMP level was not different from control. In spite of increase in RNA level, the protein level of MMP9 was similar in ADOII and control and that of integrin β3 was decreased in ADOII (Fig. 3 *D*).

### The knockdown of ClC‐7 in mouse bone marrow monocytes (BMMs) reproduces the phenotype of osteoclasts from the ADOII patient

**Figure 4:**
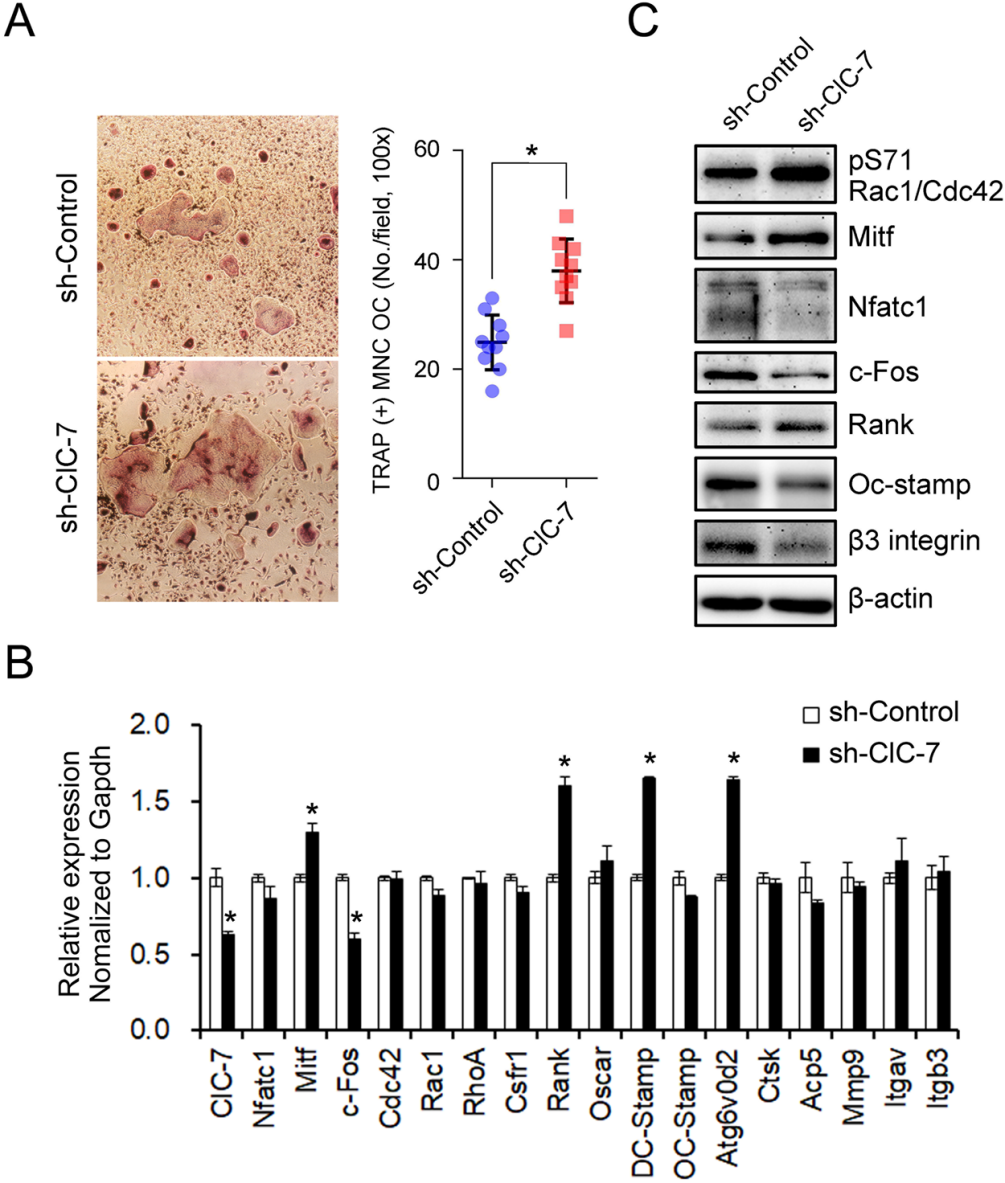
Knockdown of ClC‐7 reenacts the enhanced differentiation and molecular signature of the ADOII patient's osteoclasts. (*A*) Primary BMMs obtained from 8‐week‐old C57BL/6 mice long bone were transfected with lentiviral vectors with shRNA containing mouse ClC‐7‐specific constructs and control shRNA and differentiated into osteoclasts in the presence of 30 ng/mL RANKL and 30 ng/mL M‐CSF for 5 days. TRAP^+^ multinuclear cells (MNCs) with more than three nuclei were counted as osteoclasts. Scale bar = 100 µm. *p < 0.05 compared with control. (*B*, *C*) Expression of mRNAs and proteins for major transcription factors and marker genes for osteoclasts examined by real‐time qPCR (*B*) and Western blot analyses (*C*). Data shown are representative of three independent experiments.

To validate whether the molecular changes shown in the ADOII patient are common phenomena associated with ClC‐7 loss‐of‐function, we then conducted an in vitro ClC‐7 knockdown experiment. Mouse BMMs transfected with lentiviral shRNA targeting ClC‐7 revealed an enhanced osteoclast formation compared with control shRNA (Fig. 4 *A*). Knockdown of ClC‐7 in osteoclasts showed an enhanced mRNA expression of Mitf, Rank, DC‐stamp, and Atp6v0d2 and reduced c‐Fos compared with shControl. The RNA expression of Acp5, Mmp9, and Itgb3 that increased in the AODII patient's osteoclasts failed to show any difference in ClC‐7 knockdown osteoclasts (Fig. 4 *B*). In protein level, the Ser‐71 phosphorylation of Rac1/Cdc42 was increased in the ClC‐7‐knockdowned osteoclasts compared with control shRNA. ClC‐7 knockdown showed the increased protein expression of MITF and Rank but decrease of Nfatc1, c‐Fos, OC‐stamp, and β3‐integrin compared with control shRNA (Fig. 4 *C*). These results suggest the enhanced osteoclast differentiation in the ClC‐7‐mutated monocytes can be the result of an activation of Rac1/Cdc42 and expression of MITF and RANK.

## Discussion

In this study, we identified a patient with ADOII harboring a missense mutation, c.643G>A (p.G215R) in exon 7 of *ClC‐7* gene. The G215R mutation of *ClC‐7* gene has been known as the most common cause of ADOII.^14^, ^15^ In the process of lacunae acidification, the lysosomes containing ATPase H+ channels and ClC‐7 chloride channels in its membrane are inserted into ruffled border of osteoclasts and efflux their proton content into lacunae through ATPase H+ channel as well as chloride through ClC‐7.^16^, ^17^, ^18^ Compared with wild type ClC‐7 protein, the mutated G215R ClC‐7 fails to integrate into lysosome and is retained in the endoplasmic reticulum (ER), resulting in the insufficient expression of ClC‐7 channel in ruffled border of osteoclasts.^19^

In lysosome, ClC‐7 has a function of a chloride‐proton antiporter that affects lysosomal acidification.^7^ However, the cell membrane with dysfunctional G215R ClC‐7 revealed defects in both proton and chloride influx, leading to intracellular chloride accumulation.^16^, ^20^, ^21^, ^22^ Chloride ion can work as a ligand of multiple proteins and affects enzymatic activities of proteins, including G‐proteins and protein kinases.^23^, ^24^, ^25^, ^26^ Recent functional study of cilia showed that the disturbance of intracellular chloride gradient by the inhibition of chloride channel can affect the activation of Cdc42 GTPase that is essential for cell morphology and endocytosis.^13^, ^27^

In this study, we confirmed the enhanced Ser‐71 phosphorylation of Rac1/Cdc42 protein in the osteoclast from the ADOII patient and ClC‐7 knockdown BMMs. Ser‐71 phosphorylated Rac1/Cdc42 appear to be in their active conformation according to pull‐down assay with PAK CRIB‐domain and Rho‐GDI.^28^ Both Rac1 and Cdc42 belong to GTPases of the Rho subfamily that exert on the actin cytoskeleton as well as differentiation and function of osteoclast.^29^, ^30^ However, evidence showed that the phosphorylation of Rac1 at Ser‐71 by Akt may inhibit GTP binding of Rac1, attenuating the signal transduction pathway downstream of Rac1.^28^, ^31^, ^32^ It is quite possible that the enhanced phosphorylation of Cdc42 can be associated with the augmented osteoclast differentiation in the ADOII patient. Cdc42 is well known to have a multifunctional role during osteoclastogenesis, which promotes proliferation and differentiation in osteoclast precursors and affects polarization of mature osteoclasts.^30^ Osteoclasts with dysfunctional G215R ClC‐7 can have an increased intracellular chloride level,^20^ which can lead to the enhanced activation of Cdc42 and osteoclast differentiation.

A further interesting finding of this study is that an enhanced osteoclast differentiation in ADOII of G215R ClC‐7 is associated with augmented expression of MITF, a basic helix‐loop‐helix‐leucine zipper transcription factor.^33^ In osteoclast precursor, MITF form a complex with PU.1 in the presence of M‐CSF.^34^ In this condition, RANKL/RANK signaling increases MITF expression itself as well as its phosphorylation through p38 MAP kinase.^35^ Cdc42 also promotes phosphorylation of MITF.^30^ The phosphorylated MITF recruits co‐activators such as AP1, PU.1, and NFATc1 and binds to the promoter of target genes and initiates the target gene transcription, including *Ctsk, Oscar, ClC‐7*, and *TRAP*.^36^, ^37^, ^38^ Our data showed the activation of Cdc42 and enhanced expression of RANK in the ADOII patient and it can increase the phosphorylation and expression of MITF, which can be responsible for the enhanced osteoclast differentiation in ADOII.

**Figure 5:**
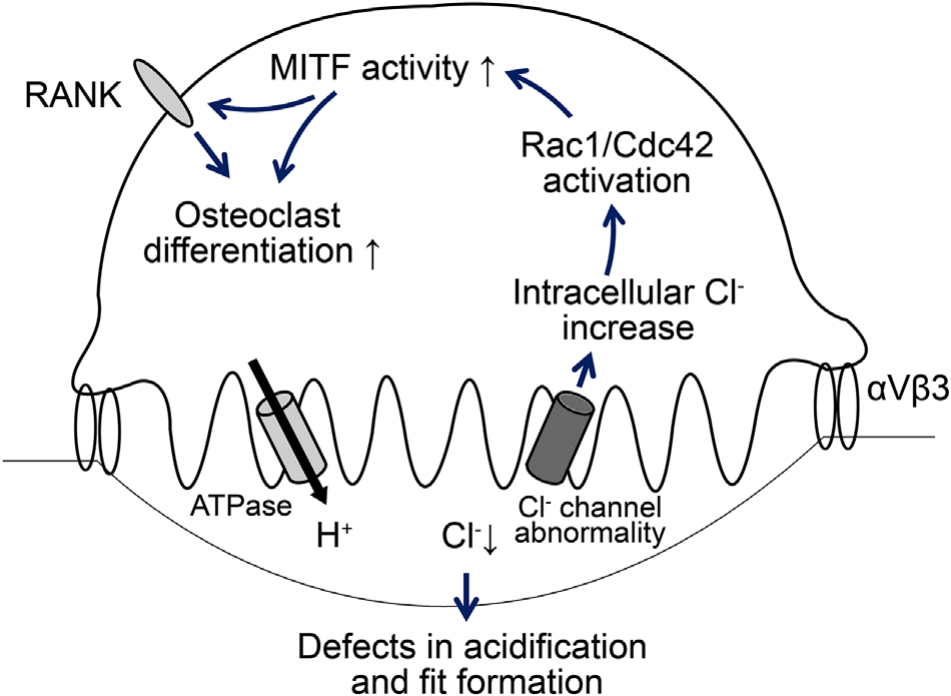
Schematic diagram drawn from this study. Defect in ClC‐7 chloride channel induces an acidification failure of resorption fit as well as accumulation of lysosomal chloride. Increased intracellular chloride can induce an activation of Rac1/Cdc42 that can be associated with enhanced osteoclast differentiation as a result of increasing MITF and RANK expression.

In conclusion, we provide a mechanical insight to understand how osteoclast differentiation can be enhanced in ADOII patients. Increased intracellular chloride by defective chloride channel can induce the activation of Cdc42 that can be associated with the enhanced activation of MITF and RANK signaling (Fig. 5). These clues found in this case report need to be further investigated to understand the role of chloride in differentiation and function of osteoclasts.

## Disclosures

All authors state that they have no conflicts of interest.

## Supporting information

Supporting Data S1.
