# Supplementary material for: Enhanced Activation of Rac1/Cdc42 and MITF Leads to Augmented Osteoclastogenesis in Autosomal Dominant Osteopetrosis Type II
Source: JBMR Plus. 2018 Jul 16;3(2):e10070. doi: 10.1002/jbm4.10070 (PMC6383696; doi:10.1002/jbm4.10070)
Supplement: Supplementary file 1 — Supporting Data S1. [file JBM4-3-na-s001.docx]

**Enhanced activation of Rac1/Cdc42 and MITF as a possible mechanism of augmented osteoclastogenesis in autosomal dominant osteopetrosis type II with G215R mutation of chloride channel 7 gene**

Youn-Kwan Jung^1^*, Ki-Tae Kwon^2^*, Ji-Ae Jang^1^, Min-Su Han^1^, Gun-Woo Kim^1,3^, Seungwoo Han^2^

^1^Laboratory for arthritis and bone biology, Fatima Research Institute, Daegu Fatima Hospital, Daegu, Republic of Korea, ^2^Department of Internal medicine, Kyungpook National University Hospital, Daegu, Republic of Korea, ^3^Department of Internal medicine, Daegu Fatima Hospital, Daegu, Republic of Korea

Supplementary table 1. PCR primers used for amplification of CLC-7 exons and real time qPCR in this study.

| Targets | Primer sequence (5' to 3') | | Length |
| --- | --- | --- | --- |
|  | Forward | Reverse |  |
| Exon 1 | CGTTGCAGGTCACATGGTC | GCCTCCGAAGACTCCAGAC | 470 |
| Exon 2 | CGGATCAGTTCTGCTTCCAG | CATGCTGTCACTGCTGTCCT | 511 |
| Exon 3 + 4 | TGCTGGGATTGTAGGTGTCA | GAGCAGCCTTCTTGGTTACG | 629 |
| Exon 5 + 6 | CACACTGGGCCCTTCATAAT | TCTGCTCCTCCTGAGGTTGT | 810 |
| Exon 7 | GTGTCTGCTGCTCTCCTCAG | GCTCCTGAACCAGCAAAGAG | 243 |
| Exon 8 + 9 | GCTTGGCTGCTGTTTAGCTC | AAGCCCATCTCCCTGAGTG | 764 |
| Exon 10 + 11 | GTGCTGACCCTGCTGTCTCT | AGGACCAAGGCCTGACAGA | 797 |
| Exon 12 | CACTGGCAAGTCCAGAGAGG | GCAGCAACTGTGTGACATCC | 559 |
| Exon 13 | CCAGTGTGTTTCTCCCCTGT | CTGTGGTTTTTGCCAACAGA | 443 |
| Exon 14 | ATTGCTCTGCTGGACACCTT | GCAGGGCCTCACTTCCTAC | 551 |
| Exon 15 | CAGTGTCCTCCATCAGGGACT | CTCTGAGATCTGGGTGGACAG | 401 |
| Exon 16 | CTCCCAACGTGTGCTCTCTC | ATCCTCCTGCCTTGGTCTCT | 306 |
| Exon 17 | TGAGAACAGGGAGCCTTCTG | AGGTGCGACACTTTTGTCCT | 432 |
| Exon 18 + 19 | GGTGACTGTGCCCTCTGC | CCCAGAAACCCTGAGCCTAC | 730 |
| Exon 20 + 21 | CTGTGAGCCTCCAAACAGC | GTCCACACAGCCCTCCAT | 717 |
| Exon 22 + 23 | AGGCTGGTGTGAGCAGGTAG | GCCCCTTGACTTCAGCTCTA | 638 |
| Exon 24 + 25 | CTGAAGTCAAGGGGCTGAGG | AGACCACTGCCCACAACAG | 806 |
| CLC-7 | TGCTCATGACCGCCAAGAT | AGTGCAGGAAGGGCACACTCT | 90 |
| NFATc1 | GCGAGCCGTCATTGACTGT | CGATGTCCGTCTCTCCTTTCC | 80 |
| MITF | CGGGTCTCTGCTCTCCAGATT | AAGGAGGTCTTGGCTGCAGTT | 80 |
| FOS | AGGAGAATCCGAAGGGAAAGG | TGTCTCCGCTTGGAGTGTATCA | 90 |
| CDC42 | GCCTCCAGAACCGAAGAAGA | CGACACCAGCTGTGCAGAAA | 80 |
| RAC1 | TGCATTGTTGTGCCGAGAAC | GAGCAAGTGTCTGCACCTCCTA | 100 |
| RHOA | TGAGTCACCACTTCAGGGCTTT | AGCTGCATGAACTTGGGCTTT | 110 |
| CSF1R | CCTCCTCTGGGAGATCTTCTCA | GGCCATTTGGTATCCATCCTT | 100 |
| RANK | ACGCTGGCCTGCTTTACGTA | CATGTTTAGCCCTTTCCCAAAA | 110 |
| OSCAR | TAGCTGAAAGGAAGACGCGATT | TAGACGGCAGTGCTGGGATT | 80 |
| DC-STAMP | CGATTTTTGGGCCCTTGTG | CACAGGGCCTCTGTTGATGTC | 100 |
| OC-STAMP | GTGGACTGGGCTCAGAAGTTG | GGTTGAAGAGGAAAGGGATGAA | 100 |
| ATP6V0D2 | ATCCAACCTTCGGCAAACTCT | AATGATCCGCTACGTTCTTCATC | 90 |
| CTSK | CCAAATTTTCCAGCCGATCA | CTTCGTTTCGGCAGCAAAGT | 100 |
| ACP5 | CGTGCTAGCCGGAAACCAT | AGTTCCAGCGCTTGGAGATC | 80 |
| MMP9 | CCCTGGAGACCTGAGAACCA | CCACCCGAGTGTAACCATAGC | 80 |
| ITGAV | AGATGTTGGGCCAGTTGTTC | GCAACTCCACAACCCAAAGT | 183 |
| ITGB3 | GCTATGGTTCTCTCGCAAGG | GCTATGGATGAGGGACAGGA | 164 |
| GAPDH | AGCCACATCGCTCAGACAC | GCCCAATACGACCAAATCC | 66 |

**Supplementary Methods**

Sequencing of CLC-7 DNA

All participants provided written informed consent before blood sampling and DNA analysis. Genomic DNA was extracted from whole bloods of participants by genomic DNA extraction kit (Cosmo Genetech, Korea). The reference sequence of human CLC-7 gene was obtained from the NCBI database (NM_001287) and genomic sequence containing introns was from Human BLAT Search tool of UCSC Genomic Institute (http://genome.ucsc.edu/cgi-bin/hgBlat). All exons of the CLC-7 gene were amplified by polymerase chain reaction (PCR) using primer pairs corresponding to each exon (Supplementary Table 1). Sequencing was performed directly on PCR products after purification at qualified specialized facility (GeneAll, Korea).

In Vitro Osteoclast differentiation and pit formation assay

Peripheral blood mononuclear cells (PBMCs) isolated from whole blood by Ficoll density gradient method were cultured in complete α-MEM for 24 hr. After discarding the adherent cells, the suspended cells were cultured with recombinant human M-CSF (30ng/ml, R&D Systems, Minneapolis, MN) for 7 days to differentiate into macrophages. PBMC-derived macrophages were plated in 48-well plates at a density of 1 x 10^5^ cells per well and cultured in complete α-MEM with 30 ng/ml M-CSF and 30 ng/ml RANKL (R&D Systems) for 21 days. The formation of mature osteoclasts was assessed by TRAP activity using TRAP staining kit (Sigma-Aldrich, MO, USA) and counted TRAP positive multinucleated cells with more than 3 nuclei. At least five randomly chosen fields under the microscope (×100) were evaluated for each well, and an average score was calculated. For pit formation assay, PBMC-derived macrophages were plated on sterile dentine slice (Immunodiagnostic systems, Boldon, UK) in 48-well plates and cultured as described above. After 21 days culture, cells were removed gently with 10% bleach solution and the dentin slices were stained with 1% toluidine blue. The resorption area in triplicated dentin slices was quantified via Nikon's NIS-Elements system (Nikon, Japan). Statistical analysis of the data was performed by Student’s t-test using the GraphPad Prism software (La Jolla, CA).

Real-time quantitative PCR

Total RNA was isolated from cultured mature osteoclasts using EasyBlue RNA extraction kit (iNtRON, Korea) at day 21. For real-time qPCR, 25ng of cDNA used as template and 10ul of SYBR green master mixture (Applied Biosystems, Waltham, MA) were mixed with gene-specific primers (Supplementary Table 1) in a total volume of 20ul for each reaction. All samples were run in triplicate and normalized to GAPDH. The calculation of the relative expression was performed using the 2^-∆∆^CT method.

Western blot analysis

Cell lysates from cultured mature osteoclasts were separated by SDS-PAGE and transferred onto a PVDF membrane. After blocking with 5% skim milk, immunoblotting was performed using anti- c-Fos (#2250), p-Rac1/Cdc42 (Ser71) (#2461), MITF (#12590), ITGB3 (#4702) from Cell Signaling technology (Danvers, MA), NFATc1 (ab2996) from Abcam (Cambridge, UK), DC-STAMP (sc-87673) and RANK (sc9072) from Santa Cruz Biotechnology (Dallas, TX), MMP9 from (MAB936) from R&D Systems, and OC-STAMP (MBS540393) from MyBioSource (San Diego, CA). The membranes were washed with TBS-T and incubated with HRP-conjugated secondary antibody (Santa Cruz Biotechnology).

*ClC-7* shRNA knockdown and osteoclast differentiation in mouse bone marrow macrophages (BMMs)

Primary bone marrow cells were obtained from 8-week-old C57BL/6 mice long bone. After incubation at 37℃ for 1 day, non-adherent monocytes were cultured with complete α-MEM in the presence of 30ng/ml of M-CSF for 1 day and then infected with 1 x 10^5^ infectious unit (IFU) of mouse *ClC-7*-specific shRNA lentiviral particles (Santa Cruz technology, #sc-42390-V) or scramble shRNA lentivirus particles as control (Santa Cruz technology, #sc-108080) with 4 ug/ml of polybrene (Santa Cruz technology, #sc-134220) and 30 ng/ml of M-CSF overnight. After an additional 1 day culture, BMMs were cultured with 30 ng/ml of M-CSF and 30 ng/ml of RANKL for 5 days to differentiate mature osteoclasts. Osteoclasts differentiation was analyzed by TRAP activity staining and effects of *ClC-7* knock-down were analyzed by western blot and RT-qPCR.

Infection of Raw264.7 cells or

BMMs with recombinant lentivirus was conducted in the

presence of 8 mg/ml polybrene (Sigma–Aldrich, St. Louis,

MO) for 16 h, infected cells were selected in 3.5 mg/ml puro-

mycin for 3–5 days.

Infection of Raw264.7 cells or

BMMs with recombinant lentivirus was conducted in the

presence of 8 mg/ml polybrene (Sigma–Aldrich, St. Louis,

MO) for 16 h, infected cells were selected in 3.5 mg/ml puro-

mycin for 3–5 days
